# The interaction of excited atoms and few-cycle laser pulses

**DOI:** 10.1038/srep34101

**Published:** 2016-09-26

**Authors:** J. E. Calvert, Han Xu, A. J. Palmer, R. D. Glover, D. E. Laban, X. M. Tong, A. S. Kheifets, K. Bartschat, I. V. Litvinyuk, D. Kielpinski, R. T. Sang

**Affiliations:** 1 Australian Attosecond Science Facility and Centre for Quantum Dynamics, Griffith University, Brisbane, Queensland 4111, Australia; 2 Institute for Atomic and Nuclear Physics, University of Liege, Liege 4000, Belgium; 3 Graduate School of Pure and Applied Sciences, and Center for Computational Science, University of Tsukuba, Tsukuba, 305-8571, Japan; 4 Research School of Physics and Engineering, The Australian National University, Canberra ACT 0200, Australia; 5 Department of Physics and Astronomy, Drake University, Des Moines, IA 50311, USA

## Abstract

This work describes the first observations of the ionisation of neon in a metastable atomic state utilising a strong-field, few-cycle light pulse. We compare the observations to theoretical predictions based on the Ammosov-Delone-Krainov (ADK) theory and a solution to the time-dependent Schrödinger equation (TDSE). The TDSE provides better agreement with the experimental data than the ADK theory. We optically pump the target atomic species and measure the ionisation rate as the a function of different steady-state populations in the fine structure of the target state which shows significant ionisation rate dependence on populations of spin-polarised states. The physical mechanism for this effect is unknown.

Recently, there has been much interest in the generation and utilisation of few-cycle light pulses that have a length of three or even less optical cycles. This interest is in no small part due to the possibilities in applications such as lightwave electronics^1^,^2^, high-order harmonic generation^3^, above-threshold ionisation, and multiple ionisation^4^. Additionally, the precise control of the carrier envelope phase (CEP) of a few-cycle laser pulse in strong laser-matter interactions opens many possibilities^5^,^6^,^7^. All these effects share a common starting point, namely, the strong-field ionisation of an atom.

Strong-field atomic ionisation is a highly nonlinear process that has been realised through high laser intensities obtained by tightly focusing a few-cycle pulse of light with a high peak pulse power^8^. The different interaction regimes that few-cycle light-matter interactions can be characterised by depend on the magnitude of the electric field in the interaction region relative to the ionisation potential of the atom. In the first regime, the electric field is strong enough to induce a perturbative non-linearity in the matter, but not strong enough to cause significant ionisation of atoms. In the second regime, the electric field is sufficiently strong to provide a high probability of ionisation in the target material. This is known as the strong-field regime. The Keldysh parameter, 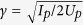, is used to determine what regime a particular interaction belongs to. Here *I*_*p*_ is the ionisation potential of the medium and *U*_*p*_ is the ponderomotive energy, i.e., the kinetic energy imparted to an ionised electron by a linearly polarised oscillating electric field^9^. The perturbative regime corresponds to *γ *> 1 and the strong-field regime to *γ* < 1^8^.

In the strong-field regime, it is possible to treat the light-matter interaction semi-classically through a three-step model^10^ that describes the effects and results of the interaction. The first step corresponds to ionisation by the light pulse as a result of the suppression of the Coulomb atomic potential. The second step involves the acceleration of the electron wavepacket by the electric field of the light pulse. The motion of the ionised electron corresponds to the classical motion of a charge in an oscillating electrical field, which then imparts ponderomotive energy to the ionised electron. The third step may include a recollision of the electron, which results in a variety of interactions with the parent ion.

Inelastic recollision can result in secondary electron promotions within the parent ion, either causing a direct secondary ionisation known as non-sequential double ionisation (NSDI)^4^ or exciting another valence electron to a higher energy state. This excitation lowers the effective second ionisation potential of the atom, thereby providing the opportunity for ionisation in the remainder of the laser pulse in a process known as recollision-enhanced secondary ionisation (RESI)^11^. The study of NDSI and RESI provides enlightening information about the electron dynamics of an ionising system. Another possible interaction is the recombination of the wavepacket with the parent ion and the subsequent creation of a photon that has a harmonic frequency of the driving field. This process is known as high-order harmonic generation (HHG)^8^ and is being studied as a potential method to create a tabletop XUV laser source. Elastic collisions may also occur, or the trajectory of the returning electron may not intersect with the parent ion which provides above-threshold ionisation (ATI) electrons^12^.

As all these processes depend upon the initial ionisation, it is vital to have a good understanding of this process. The most common theoretical method used to describe the process is based on the work of Ammosov, Delone, and Krainov. It is commonly known as the ADK theory^13^ and makes two essential assumptions, namely: 1) Only the initial and final wavepackets of the electron are relevant in the ionisation process. 2) The energy of a single photon is not sufficient to promote the valence electron into the continuum state, nor is the electric field of the peak high enough to suppress the atomic potential barrier sufficiently to release the valence electron to the continuum. The ADK theory is based on an analytical expression for the tunnel ionisation rate that was derived by Perelomov *et al*.^14^. In atomic units, which are in use throughout this paper except where indicated otherwise, it is given by


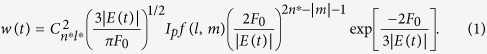


Here |*E*(*t*)| is the electric field of the laser pulse, n^*^ is the effective principle quantum number, l^*^ is the effective orbital angular quantum number, *m* is the projection of the angular momentum quantum number, *I*_*p*_ is the ionisation potential of the target species, 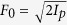, and 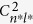 is a dimensionless constant that is unique for the atomic system under consideration. Approximating a solution for 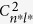 was the purpose of the work done by Ammosov *et al*. Finally, the term *f*(*l*, *m*) is given by


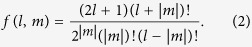


The ADK theory is not valid in the intensity regime for over-the-barrier ionisation (OBI). There have been several attempts to rectify this shortcoming. One method involves correcting the wavefunction of the ejected electron for the Coulomb potential^15^, thereby accounting for the possibility of OBI^3^. Another modification to account for OBI involves examining the ionisation rates across a broad range of atomic species, and then using the data to apply an empirical correction to the ADK formula^16^.

Despite the limitations of ADK-based methods for calculating the ionisation rate, they are attractive to utilise since they are computationally far less expensive than attempting to find solutions of the time-dependent Schrödinger equation (TDSE) for the ionising system. This has made ADK modelling the traditional method until the past decade, when several techniques to obtain approximate solutions of the TDSE were developed (see, for example^17^,^18^ and references therein). These techniques are taking advantage of significant increases in computational power and available resources.

The aim of the present work is to investigate the strong-field ionisation from atoms in an excited state, for which there have been very few experimental investigations to date. Experiments have been conducted to investigate the strong-field ionisation of Li^19^ (*γ* = 0.09 to 0.21). That work, however, focusses on identifying the role of intermediate excited states in the Li atom during the ionisation process, rather than considering ionisation from an initially excited atomic state as will be presented here. Experiments examining the ionisation of metastable xenon have been performed by Huismans *et al*.^20^ using a *λ* = 7 *μ*m laser capable of providing pulses in the picosecond regime in order to examine holography between directly ionised and rescattered electron wavefunctions (*γ* = 0.8 to 1.5). Our work uses much higher laser intensities relative to the ionisation potential of the initial state than the work performed in ref. 20 and investigates different ionisation regimes. Recent experiments conducted by the authors demonstrate significant differences in the transverse electron momentum distribution for the OBI regime compared to the tunnelling regime^21^.

Singly excited states of noble-gas atoms have an electron in the valence shell, which leaves a hole in the remaining electron core. The *jj* angular-momentum coupling scheme describes these states. However, *LS* coupling notation suitably describes the 2*p*^5^(^2^*P*_3/2_)3*s*^3^*P*_2_ state of neon (hereafter defined as Ne^*^) that we investigate in this work^22^. Ne^*^ is forbidden by selection rules to optically decay via single-photon dipole-allowed transition to the ground state. It has a lifetime of approximately 14 seconds and has been previously used in laser cooling/atom trap experiments^23^,^24^,^25^,^26^, due to an accessible closed cooling transition to the ^3^*D*_3_ state at 640.24 nm. Below we present an experimental investigation of strong-field ionisation of Ne^*^. Note that neon has a second metastable state (^3^*P*_0_) which is not considered in this work.

Investigating the strong-field ionisation of a metastable noble-gas species is interesting for several reasons. Firstly, the ionisation potential of Ne^*^ is only 5.1 eV, and hence it is relatively straight forward to investigate OBI phenomena with laser systems that are routinely used in strong-field physics experiments. Noble-gas species have closed single-photon dipole-allowed transitions that can be used to manipulate the trajectories of the atoms as well as optically pump the target atom. It is therefore possible to investigate the role of the initial atomic state in the strong-field ionisation process. For example, it is possible to spin-polarise the target atom and investigate ionisation dynamics from an oriented atomic system.

Describing strong-field ionisation experiments is also a challenge to theory. The critical field at which the unperturbed atomic energy level lie above the potential barrier and hence OBI becomes possible is given by 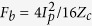
16. For Ne^*^, this corresponds to a laser intensity of 2.7 × 10^12^ W/cm^2^, which is relatively low compared to the maximum available in our experiment. Consequently, our experimental regimes can easily be varied from the case where tunnelling ionisation is dominant to the case where OBI is the prevalent process. This provides data from a challenging target over a wide range of experimental parameters and facilitates an extensive test of our current theoretical understanding of strong-field physics.

We present a new experimental apparatus that is capable of performing an experimental investigation of the strong-field ionisation of Ne^*^. We compare the measured ionisation data to predictions from the ADK and the TDSE theories. We also present first results for the ionisation of optically pumped Ne^*^ and investigate the role of the initial atomic state in strong field ionisation.

## Results

The experiment was prepared as described in the Methods section. In order to examine the response of spin-averaged Ne^*^ to ionisation intensity a number of data runs were performed at different laser intensities with Keldysh parameters ranging from *γ* = 0.37 to 2.32. The experimental parameters were as follows. The integration time of the experiment was 120 s. The laser pulses had random CEP and a pulse length of 6.3 ± 0.2 fs, measured as the full width at the half maximum of intensity. The final Ne^*^ ion yield (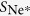) was determined according to 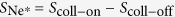, where *S*_coll−on_ is the time-of-flight (TOF) measurement with the optical collimator on, and *S*_coll−off_ is the TOF measurement with the optical collimator off. *S*_coll−off_ contains ionisation information from all atomic states in the beam, while *S*_coll−on_ contains information on an atomic beam with an enhanced Ne^*^ flux. This results in ion yield information that is provided solely by the enhanced number of Ne^*^ atoms in the atomic beam. Background contributions in all measured cases were less than 0.6% of the signal.

**Figure 1:**
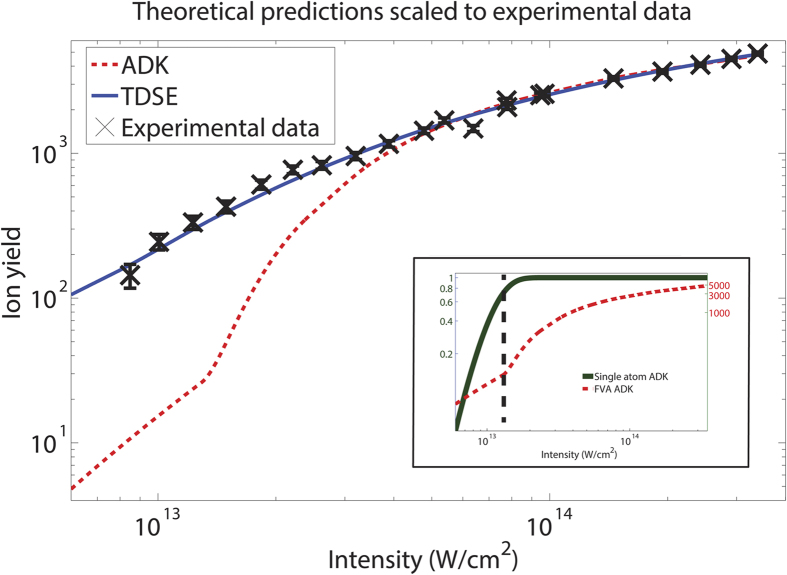
Comparison of experimental data with theoretical predictions. The theories are scaled using a spline fitting procedure. For the ADK fit, *A* = 0.18 and *η* = 3.89, with *χ*^2^ = 0.41. For the TDSE fit, *A* = 0.42 and *η* = 1.59, with *χ*^2^ = 0.25. Inset: A comparison of the probability of ionisation for a single atom using the ADK theory against the focal volume averaged (FVA) ADK application as described in the main plot. It is observed that the sharp change in ionisation rate at ~1.5 × 10^13^ W/cm^2^ observed in the FVA data corresponds to the point where the ionisation probability of a single atom is approximately 80%. The black dashed line is provided as a guide for the eye.

The theoretical results were obtained as described in the Methods section. In order to compare the predictions to experiment, the theoretical results were scaled to fit the experimental intensity dependence using a Matlab two-parameter spline fitting procedure. The scaling was done for both the ion yield and the laser intensity using the equation *y* = *A* × *spline*(*ηx*). Here *spline* is the spline function that is fit to the theoretical predictions, *A* is the ion yield scaling factor, and *η* is the laser intensity scaling factor. The method has been used in previous work to compare theory to experiment in the case of atomic hydrogen^27^,^28^. The uncertainty presented in the experimental section is given by the Poissonian counting error. Uncertainties in the laser intensity calibration include measurement error as well as systematic power measurement to intensity calculation errors. The latter is corrected with the intensity scaling. The experimental results and theoretical comparison are shown in Fig. 1.

It should be noted that there appears to be an outlying data point below the curve at 6.38 × 10^13^ W/cm^2^. The five data points at 6.38, 7.76, 7.79, 9.46 and 9.70 × 10^13^ W/cm^2^ were taken by employing two different experimental techniques for intensity variation; one where the intensity was controlled solely by adjusting the half-wave plate and the germanium plates, and one where the intensity was locked at the germanium plates while flip-in pellicle beamsplitters were used to reduce intensity. This was done to determine the accuracy of overlap between the two experimental techniques. For the point in question it was ascertained that using the half-wave plate for intensity control at this intensity would not effectively maintain the polarisation state of the light and it is likely an outlier caused by a systematic error due to this issue.

**Figure 2:**
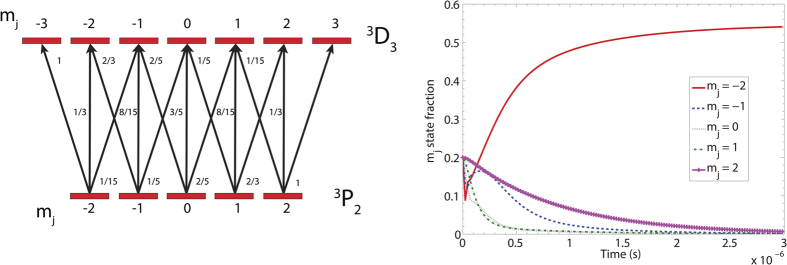
The panel on the left shows the optical pumping transitions for the ^3^*P*_2_ → ^3^*D*_3_ states with the associated magnetic substates, also shown are the decay rates for the individual sublevel transitions. The panel on the right displays the time evolution of the *m*_*j*_ states of ^3^*P*_2_ neon being pumped with *σ*^−^ polarised light tuned to the ^3^*D*_3_ → ^3^*P*_2_ transition. The intensity of the light is 20 times the saturation intensity of the transition. These results describe the system reaching steady state as described in the text, with 50% of the atoms in the displayed ^3^*P*_2_ *m*_*j*_ = −2 state. The remainder exist in the ^3^*D*_3_ *m*_*j*_ = −3 excited state, which is not displayed in the figure. When the atoms leave the pump beam they decay from the excited state into the *m*_*j*_ = −2 state as described in the main text.

The present work also utilised optical pumping of the target atom with another laser beam tuned to the cooling transition in order to spin-polarise the target atom. If the optical pumping laser light is circularly polarised, it acts on a target atom by causing many single-photon absorptions followed by relaxations due to spontaneous emission. The result of this process is that the atomic population is transferred into the largest *m*_*j*_ = ±2 states (+2 for *σ*^+^ left hand circularly polarised light and −2 for *σ*^−^ right hand circularly polarised light) after the interaction with the light beam^29^. Atoms with these magnetic projection quantum numbers have the maximum total angular momentum and are spin-polarised. The sublevel transitions and their associated decay probabilities are shown in Fig. 2. An additional laser beam was added after the optical collimator to facilitate the optical pumping. The laser beam interacted perpendicular to the atomic beam and was on resonance with the cooling transition used in the optical collimator. The laser beam was retro-reflected and the laser detuning is set to 0 MHz so that the net scattering force on the atoms in the atomic beam was zero^30^, thus ensuring that the trajectory of the atoms remained unaltered, which avoided a loss in ion yield signal. The polarisation state of this beam was altered using a quarter-wave plate and facilitated a polarisation change which changed the distribution of *m*_*j*_ states. The optical pump beam has a measured power of 125 mW, across a collimated beam geometry with a 6.1 mm radius. This gives a pump intensity of 20 times the saturation intensity (4.22 mW/cm^2^) of the optical transition. We modelled the optical pumping process by numerically evaluating the optical Bloch equations (OBEs) in the rotating-wave approximation (RWA). The OBEs fully describe the evolution of the internal atomic states in the presence of an external field including the atomic state coherences and spontaneous decay. For example, Fig. 2 shows the evolution of a Ne ^3^*P*_2_ atoms pumped by *σ*^+^ light. The system reaches a steady state after approximately 1 *μ*s with 50% of the atoms in the ground ^3^*P*_2_ *m*_*j*_ = 2 state and 50% of the atoms in the excited ^3^*D*_2_ *m*_*j*_ = 3 state. A fully polarised state is only reached after a period of relaxation where the system is allowed to evolve without the influence of the pump laser. This second step takes a further 80 ns, after which approximately 99% of the atoms are in the desired ^3^*P*_2_ *m*_*j*_ = 2 state. On average, an atom was under the influence of the optical pumping beam for 12 *μ*s, which is more than sufficient to fully polarise the atomic beam. Between the optical pumping region and the interaction region (approximately 45 cm), there is a small residual magnetic field from the Earth, which could have induced a small depolarisation of the atoms^31^. However, our results show that the majority of atoms remain well polarised.

**Figure 3:**
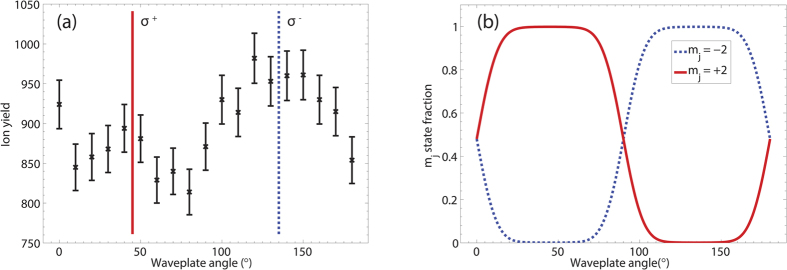
(**a**) Measurements of ionisation yield as a function of the angle that the quarter-wave plate makes relative to the polarisation axis defined by a linear polariser when using 640.24 nm optical pumping light. The intensity of the ionising laser is *I* = 9.2 × 10^13^ W/cm^2^. This intensity was chosen as it allows accurate use of ADK modelling of the ion yield for later analysis (see Fig. 1). The pump light is intended to pump the atom beam into an ensemble of different *m*_*j*_ states, depending on the alignment of the fast axis of the quarter-wave plate with respect to a linear polariser. There is a significant ion yield difference between the Ne^*^ beam being pumped with *σ*^±^ circularly polarised light and being pumped with linearly polarised *π* light. This indicates an average ionisation potential difference between a spin-polarised atomic ensemble compared to a spin-averaged atomic ensemble. (**b**) Indicates the expected *m*_*j*_ state fraction of the beam at different wave-plate angles. When pumped with *π* light, the state distribution for all remaining states are approximately 0.02. The modelling was performed for the experimental pump beam parameters by numerically solving the OBEs and is provided as a guide for the eye.

Figure 3 shows the ion yield when rotating the quarter-wave plate of the optical pump light. The degree of circular polarisation of the optical pump light was measured by measuring the Stokes parameters utilising the classic method involving a linear polariser and a quarter-wave plate as described in ref. 32; in this case the normalised parameter *S*_3_/*S*_0_ describes the prevalence of *σ*^+^ circularly polarised light to *σ*^−^ circularly polarised light. The average maximum absolute value for |*S*_3_/*S*_0_|_*max*_ was measured to be 0.96 ± 0.02, i.e. the maximum degree of circular polarisation is 96% in this experimental setup. Figure 3(b) is a broad function around the point of full circular polarisation and such a change in the degree of circular polarisation will have a negligible effect on the optical pumping with a less than 1% change in the fully spin polarised state. A further consistency check for the ionisaton rate as a function of optical pumping was made by flipping the quarter wave plate so that the opposite handedness for the ionisation rate could be measured with the results consistent with what is presented in Fig. 3(a). Figure 3 shows that as pump light becomes more circularly polarised, there is a corresponding increase in the ionisation rate. There are a number of important observations that can be made about this measurement. The change in the ellipticity of the optical pumping beam changes the atomic state distribution of the Ne^*^ atoms, and we clearly observe an ionisation dependence on the initial state of the Ne^*^ system. The second observation is that the ionisation rate maximises for the fully spin polarised states compared to the mixed state case created by *π* polarisations which produces a mixed distribution between all *m*_*j*_ substates. There also appears to be an asymmetry in the ionisation distribution. These are remarkable features and clearly demonstrates that the tunnel ionisation rate depends on the fine structure population of the excited state with maximum ionisation rates when the atoms are spin polarised.

## Discussion

The results exhibited in Fig. 1 are the first using Ne^*^ as a target atom, and the first to examine the strong field ionisation effects of a metastable target. The results are fit with arbitrary scaling. The ADK theoretical predictions for ion yield observe a significant increase at an intensity of 1.5 × 10^13^ W/cm^2^. This is due to the focal volume averaging used to determine the total ion yield as described in the methods section. At intensities below the sharp increase in ionisation rate, the probability of ionisation of a single atom is below unity as predicted by ADK theory and as such there is a limited number of ionisation events occurring across the entire focal volume of the laser pulse. In this region, the peak intensity of the laser pulse in the single atom ADK calculations is what limits the maximum calculated ionisation yield. At intensities above 1.5 × 10^13^ W/cm^2^, the probability of a single atom ionising is close to, or at, unity. There is a considerable increase in ion yield at this point. As the peak intensity increases, more of the outlying pulse volume contributes to the ionisation rate. In this region, the localised intensities of the laser pulse throughout the focal volume is what limits the maximum calculated ionisation yield. This is demonstrated in the inset of Fig. 1, where the ionisation probability of a single atom is compared to the ADK theoretical results that have been focal volume averaged.

There is poor agreement with the ADK fit below 4.0 × 10^13^ W/cm^2^. This is expected as ionisation due to multiphoton processes and OBI are not predicted with ADK theory, both of which may contribute to the total ion yield. This compares favourably to previous results^33^,^34^, where it was noted that multiphoton processes affect the accuracy of ADK ionisation rates at similar peak intensities. The OBI intensity for our target is below the intensity where ADK theory fails, unlike the targets used in refs 33,34. This indicates that OBI contributes to the difference between ADK and experiment at intensities below 4.0 × 10^13^ W/cm^2^, which can be confirmed through the measurement of the transverse electron momentum distribution as shown in ref. 21. As the TDSE solution accounts for both OBI and tunnelling ionisation effects, one might expect to see a better scaled fit at those lower intensities. This is indeed what we observe. At intensities higher than 4.0 × 10^13^ W/cm^2^ the fit after scaling is similar for both ADK and TDSE theories. This is expected, despite the inability of the ADK approach to model OBI ionisation. At these high peak intensities, the probability of ionisation becomes unity for both theories across a similar and large fraction of the volume of the interaction region, with focal volume averaging effects at the edges of the laser beam volume (where the probability of ionisation is less than unity in the modelled lower electric field amplitudes) causing slight differences in modelled ion yield. Physically this implies that at these high intensities, the exact process of ionisation for determining ion yield is irrelevant, as the ionisation event will always occur. For this reason, more relevant comparisons of theory should be made at intensities below 4.0 × 10^13^ W/cm^2^.

The mechanism for the modulation in the ion yield as the quarter wave plate is rotated, and hence the fine structure population distribution in the target atom state changes, as displayed in Fig. 3 is unknown. However, it may be a result of the magnetic field of the strong field pulse inducing a spin flip in the metastable state and facilitating a decay to the neutral ground state of the neon atom. In this case the ground state has a significantly larger ionisation potential and hence a change in the ionisation rate would be expected. This is the first demonstration of the effect of the fine structure on tunnel ionisation rates and this system provides a new challenge for modelling tunnel ionisation for atomic systems.

We have performed the first strong-field ionisation experiment with excited-state neon atoms and measured the complete ion yield from the ionisation of Ne^*^ atoms using a COLTRIMS setup. Our work showed that solving the TDSE, even with the necessary approximations to make the problem computationally tractable, provides better agreement with experiment than the ADK theory. This is likely the result of applying the theories to an atom with such a low ionization potential, where the basic assumptions for ADK become invalid. A maximum difference of 16% in the ion yield was experimentally demonstrated between atoms pumped to the stretched *m*_*j*_ states compared to atoms pumped into a mixture of *m*_*j*_ states using *π* polarised light.

## Methods

### Experimental setup

Few-cycle light pulses were provided by a commercially available chirped pulse amplification system (Femtopower Compact Pro CE Phase). The final laser output in typical operating conditions was a 1 kHz train of pulses 6 fs long, with a pulse energy of approximately 450 *μ*J.

The pulses from the laser system passed through a half-wave plate and a pair of germanium plates at Brewster’s angle in order to provide variable intensity from 150 mW down to 8 mW. In order to preserve the polarisation state, a series of flip-in pellicle beamsplitters were used to reduce the laser intensity further. For intensity calibration purposes, a removable quarter-wave plate was also placed in the beam path. The few-cycle pulses were then focussed into the interaction region of the detection system. The intensity of the focussed light beam in the interaction region was determined for a number of measured input powers, utilising the approach outlined in the work of Alnaser *et al*.^35^. This method provided an absolute intensity accurate to within 50%. These data are used to create a calibration curve that maps the measured power to effective intensity. In addition, this calibration curve allowed for the calculation of the beam waist at the focus, assuming a Gaussian beam propagation. The calculated beam waist diameter is 14 ± 1 *μ*m, with an associated Rayleigh range of 810 ± 120 *μ*m. The random shot-to-shot uncertainity of the laser intensity is dependent upon the uncertainity of the Thorlabs S310C power meter used to measure pulse power. This was estimated to be 11% based on manufacturer specifications.

The detection system was a cold target recoil ion momentum spectroscopy (COLTRIMS) device. This is an ultrahigh vacuum (UHV) system that utilises electric fields to separate the products of a light-atom interaction based on charge polarity^36^. The charged products are first guided onto multichannel plates to amplify the signal, and then onto delay-line detectors that record the time and location of the ion strikes on the detector. Electron momentum spectroscopy was available, but not required for this experiment. Mass spectroscopy of the product ions was performed by correlating TOF data from the delay line detectors. This was used to obtain ion counts from the interaction with the ionising laser. Position data can be used to determine ion momentum but was not utilised for this experiment.

A DC discharge source was used to generate the Ne^*^ atoms. This type of source is common for generating metastable noble-gas atoms and was repurposed from previous experiments^37^,^38^. Neon gas was fed at a pressure of 1.1 Torr past a cathode tip and through a liquid nitrogen cooled 250 *μ*m diameter nozzle into the evacuated (≈10^−6^ Torr) source chamber. The gas expands supersonically towards an anode skimmer, which provides collimation to the atomic beam downstream. The application of a high voltage across the two electrodes created a DC discharge in the region where the neon is expanding into the vacuum system. Electron collisions with neon atoms generate several products, including Ne^*^ at approximately 0.01% efficiency^39^.

Immediately following the skimmer was an optical collimator^31^,^40^ which was utilised in order to increase the Ne^*^ flux. The collimator consists of two pairs of elongated mirrors at right angles to each other that are placed near parallel to the direction of travel of the atomic beam. These mirrors were tilted slightly from parallel such that four incident laser beams frequency-locked close to the cooling transition for the ^3^*P*_2_ state create an angle frequency detuned two dimensional (2D) optical molasses along the path of the atomic beam. This 2D optical molasses reduces the transverse velocity component of only the ^3^*P*_2_ neon atoms and can be viewed as a collimation matter lens for the atomic beam.

**Figure 4:**
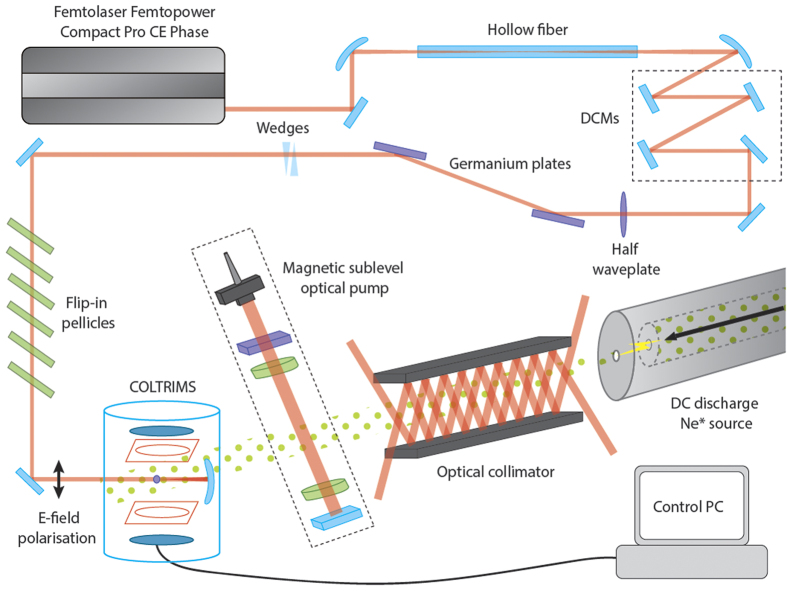
Schematic diagram of the experimental setup used in this work. Only one pair of mirrors for the optical collimator is shown, whereas two pairs are employed in the actual experiment to collimate in two directions. The optical pump laser is propagating in the same direction as the electric field of the ionising laser, which defines the quantisation axis. The magnetic sublevel optical pump apparatus was not used for the results displayed in Fig. 1.

Following the collimator, two chambers separated by two 1.5 mm apertures were used to create a differential pumping section in order to match the vacuum pressure to the COLTRIMS UHV. The first chamber contained electron deflector plates to remove charged particles from the atomic beam created by the discharge. The second chamber contained a Faraday cup that is used to measure the beam flux and assisted in aligning the Ne^*^ source. A pneumatic gate valve separated the Ne^*^ beamline from the COLTRIMS chamber. Also on this chamber were a pair of optical viewports which allowed for the atomic beam to be illuminated perpendicular to the atomic beam by two retro-reflecting laser beams at 640.24 nm, which are produced by a dye laser frequency locked and on resonance with the cooling transition. A linear polariser and two quarter-wave plates were used to alter the ellipticity of the pump beam in order to pump the atoms into various *m*_*j*_ states. When the atomic beam rached the interaction region of the COLTRIMS device, it had a diameter of 1.5 ± 0.3 mm, as measured by scanning the strong-field laser beam focus across the atomic beam and observing the change in ion yield. See Fig. 4 for a schematic of the experiment.

### Modelling the ion yield

**Figure 5:**
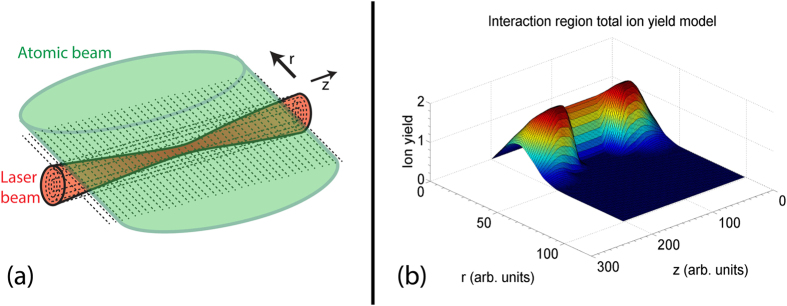
Part (**a**) is a schematic visualisation of the interaction region of the COLTRIMS. The atomic beam is travelling in the plane made with the z-axis and the *θ* = 0 angular coordinate. As the system is solved symmetrically in *θ*, the axis along the *θ* = 0 coordinate is labelled the *r* axis as the solution requires knowledge of the displacement along the radial coordinate. The laser beam is propagating in the *z* direction. Part (**b**) is a modelled 2D ionisation yield map for Ne^*^ interacting with a laser pulse with the following parameters: *I*_*pk*_ = 9.6 × 10^13^ W/cm^2^; *w*_0_ = 7.25 *μ*m; *T*_*pul*_ = 6.3 fs; atomic beam width = 1.5 mm; average atomic beam speed = 1000 m/s; atomic beam flux = 1.4 × 10^14^ atoms/sr/s. These parameters, with the exception of *I*_*pk*_, were held constant throughout the modelling.

In order to provide comparison to theory, a 3D focal-volume-averaged model was created and implemented through Matlab. It is important to correctly model the interaction region, since the low ionisation potential of metastable neon causes the ionisation probability to quickly reach unity at the centre of the pulse at relatively low intensities. This implies that, as the pulse intensity increases, the outer areas in the interaction region significantly contribute to the total ion yield when compared to the ionisation of ground-state neon. The model made the assumptions that the laser pulse was Gaussian, the divergence of the atomic beam was negligible over the interaction region, the laser pulse was completely linearly polarised, and all ions generated by the interaction were detected by the COLTRIMS. Smoothing functions based on the work of Kielpinski *et al*.^28^ were employed. A representation of the interaction region is displayed in Fig. 5, with axes labelled according to a cylindrical coordinate system.

In order to perform the focal-volume averaging, the cylindrical symmetry of the region was exploited by flattening the interaction region onto a 2D area, as shown in Fig. 5. The total ion yield for a single pulse is obtained by integrating the ion yield map displaying in Fig. 5(b), which is then multiplied by the total pulse number to give a final ion yield result.

In order to generate a curve of ion yield as a function of intensity, a batch script was designed that creates a number of input peak laser intensity *I*_*pk*_ values. The script ran the ion yield script for each value of *I*_*pk*_ and generated a plot when the batch script was completed. Two theoretical ion yields as a function of intensity plots were created for Ne^+^ ions. One curve is generated by utilising ADK theory to provide the ionisation probability as provided in Eq. (1). Values for 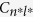 were calculated by determining the wavefunction, Ψ^*m*^, and the orbital energy of the Ne 3*s* atom^41^, before fitting to the expression^42^.


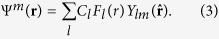


Here *F*_*l*_(*r*) is the wavefunction in the asymptotic region where tunneling occurs, and *Y*_*lm*_ are spherical harmonics.

Theoretical predictions for ionisation based on solving the TDSE are processed in the same manner. The ionisation probabilities of the Ne 3*s* orbital were calculated by solving the TDSE under the single-active electron approximation with the second-order split-operator method in the energy representation^17^,^43^. The model potential^44^ was calculated by using density functional theory with a self-interaction correction^41^. The calculated atomic ionisation potentials were in good agreement with the measured ones. The numerical convergence was cross-checked by comparing the ionisation probabilities obtained from the integration of the ATI spectra and the survival probability of the 3*s* orbital as well as the excitation to other bound states. The two results agree within a few percent.

## Additional Information

**How to cite this article**: Calvert, J. E. *et al*. The interaction of excited atoms and few-cycle laser pulses. *Sci. Rep.* **6**, 34101; doi: 10.1038/srep34101 (2016).
